# Very low oral exposure to prions of brain or saliva origin can transmit chronic wasting disease

**DOI:** 10.1371/journal.pone.0237410

**Published:** 2020-08-20

**Authors:** Nathaniel D. Denkers, Clare E. Hoover, Kristen A. Davenport, Davin M. Henderson, Erin E. McNulty, Amy V. Nalls, Candace K. Mathiason, Edward A. Hoover

**Affiliations:** 1 Department of Microbiology, Immunology, and Pathology, Prion Research Center, College of Veterinary Medicine and Biomedical Sciences, Colorado State University, Fort Collins, Colorado, United States of America; 2 AstraZeneca Inc., Waltham, Massachusetts, United States of America; 3 Department of Biochemistry, School of Medicine, University of Utah, Salt Lake City, Utah, United States of America; National Institute of Allergy and Infectious Diseases, UNITED STATES

## Abstract

The minimum infectious dose required to induce CWD infection in cervids remains unknown, as does whether peripherally shed prions and/or multiple low dose exposures are important factors in CWD transmission. With the goal of better understand CWD infection in nature, we studied oral exposures of deer to very low doses of CWD prions and also examined whether the frequency of exposure or prion source may influence infection and pathogenesis. We orally inoculated white-tailed deer with either single or multiple divided doses of prions of brain or saliva origin and monitored infection by serial longitudinal tissue biopsies spanning over two years. We report that oral exposure to as little as 300 nanograms (ng) of CWD-positive brain or to saliva containing seeding activity equivalent to 300 ng of CWD-positive brain, were sufficient to transmit CWD disease. This was true whether the inoculum was administered as a single bolus or divided as three weekly 100 ng exposures. However, when the 300 ng total dose was apportioned as 10, 30 ng doses delivered over 12 weeks, no infection occurred. While low-dose exposures to prions of brain or saliva origin prolonged the time from inoculation to first detection of infection, once infection was established, we observed no differences in disease pathogenesis. These studies suggest that the CWD minimum infectious dose approximates 100 to 300 ng CWD-positive brain (or saliva equivalent), and that CWD infection appears to conform more with a threshold than a cumulative dose dynamic.

## Introduction

Chronic wasting disease (CWD) is a transmissible, fatal, neurodegenerative prion disease affecting wild and captive cervids [1] and is now recognized in 26 states within the United States, as well as Canada, Europe, and Asia [2–4]. Evidence thus far suggests that CWD is transmitted horizontally through direct or indirect oral and/or aerosol exposure to prions shed by infected cervids [5–7], although there is evidence for vertical and/or perinatal infection [8–10]. The facile transmission of CWD continues despite exposure of cervids to very low concentrations of prions shed in secretions and excretions of infected animals [11–18]. Some explanations for this apparent enigma could include: (a) the infectious prion dose is low, (b) that excreted prions may have enhanced infectivity, and/or (c) that repeated low dose prion exposure is cumulative.

Pinpointing the mechanism(s) of natural CWD transmission is nevertheless difficult. Experimental studies of CWD in deer have employed inoculation of CWD-infected brain homogenates at dosages containing 10 grams (g) to 10 milligrams (mg) administered intracranially, orally, or by aerosol inhalation [6, 7, 11, 19–24]. However, such doses likely far exceed those conceivably encountered in nature. Currently, the lowest oral dose of CWD-positive brain published is 10 mg, which produced a 100% attack rate [25]. Likewise, the minimum published infectious oral challenge dose of scrapie or sheep-adapted bovine spongiform encephalopathy (BSE) in sheep, appears to be 50 mg of brain tissue [26]. The minimum infectious dose of CWD prions from brain or excreta in deer, especially by a natural route (e.g. oral), is unknown.

Previous investigations have demonstrated that infectious CWD prions are present in saliva [11, 16], feces [14, 15], urine [12, 13, 18], blood [11, 27], and tissues [28, 29], and that environmental exposure alone can initiate infection [5, 30]. However, experimental demonstration of CWD prion seeding activity or infectivity in naturally contaminated soil remains elusive.

CWD studies in deer [11] and transgenic mice [13, 16, 31] have raised the question as to whether saliva may have a greater infectivity when compared with exposure to urine and feces from CWD-positive donors. Other studies have indicated that the concentration of infectious prions in saliva is near the endpoint of a CWD-positive brain homogenate titrated in cervid PrP-expressing transgenic mice; i.e. 10^−6^ [32, 33]. One hypothesis that may explain such low dose infectivity would be that prions in saliva are inherently more infectious by mucosal exposure routes. Another possibility is that repeated exposures to small quantities of shed prions, either directly or through the environmental contact, may be cumulative. Cervids encounter prions shed in saliva through grooming and grazing behavior, surely more often than they encounter prion infected brain. Finally, relatively few studies in rodent models have addressed the potential influence of prion tissue source on infectivity, a facet we address, albeit to limited extent, in the present studies.

*In vitro* prion seeding amplification assays [protein misfolding cyclic amplification (PMCA) and real-time quaking induced conversion (RT-QuIC)], while not assessing prion infectivity, make possible a rapid estimation of prion concentration [34–36]. Although these assays measure seeding activity, they have been shown to not only correlate with the prion infectivity titer by bioassay [32, 33] but to have sensitivity at or beyond the limit of animal or cell culture bioassays [37–39]. Nevertheless, the relationship between intracerebral endpoint titrations in transgenic hosts and the minimum infectious dose by a natural route in a native cervid species remains unknown.

To better model and understand natural CWD infections, we investigated whether very low CWD prion doses will induce infection and explored whether exposure to the prion origin (brain or saliva) may influence infectivity and pathogenesis. These studies have also explored what the minimum infectious dose of CWD prions in white-tailed deer might be. The results demonstrate that the minimum oral infectious dose is vastly lower than we and others have previously used to establish infection, and also suggest that the infection process conforms more to a threshold dose than to a cumulative dose dynamic.

## Materials and methods

### Animals

Two [2] groups of CWD-naïve, hand-raised, white-tailed deer were sourced through collaboration with the University of Georgia Warnell School of Wildlife and were maintained indoors at Colorado State University CWD research facility. The Colorado State University Institutional Animal Care and Use Committee prospectively approved this research (Protocol numbers: 18-8396A; 18-7969A). Prior to establishing the study cohorts, the PRNP genotypes at codon 96 for all 26 deer (n = 20 96GG/n = 6 96GS) were determined to aid in seeking as balanced a distribution ratio as possible between the two genotypes in the study cohorts.

General anesthesia for the collection of biological samples from deer were administered via intramuscular injections [ketamine (2–8 mg/kg) & medetomidine (0.1–0.2 mg/kg)]. The CWD clinical status of each deer was observed daily and formally assessed every 3 months throughout the course of the study, or more frequently (weekly to daily) as clinical disease progressed. Clinical signs associated with CWD infection were observed and recorded by two individuals experienced with CWD signs, and disease progression to study endpoint was scored by pre-established, previously published clinical criteria [40]. Study endpoint was established at Stage 3 –late, to which deer were euthanized within 3 days after being identified. One [1] asymptomatic deer died unexpectedly of undetermined cause just after 9 months post inoculation. Deer were euthanized by IV injection of pentobarbital (1 mL/4.5kg). Data acquisition for all current studies was acquired up to 27 months post-exposure.

### Inocula

The CWD-positive brain homogenate inoculum used in these studies was created by pooling sections of brain from six deer with terminal CWD infection [33]. This cervid brain pool 6 (CBP6) contained 3.33 × 10^6^ 50% lethal doses/g as determined by intracranial titration in cervid PrP transgenic mice (Tg(cerPrP)5037) [32, 33, 41]. The CWD-negative brain inoculum was confirmed negative by western blot, RT-QuIC, and mouse bioassay [33]. Inoculum doses for each study were diluted to the proper weight to volume ratio in 1X PBS for each experiment, aliquoted into appropriate doses, and frozen until use.

Studies have established that the RT-QuIC rate of amyloid formation, while not measuring infectivity, is directly related to the concentration of prions in the sample seeding the reaction [32, 33]. As saliva samples contain inhibitors that may prevent detection in the RT-QuIC assay [31, 42], we utilized iron-oxide magnetic extraction (IOME) to concentrate prions and minimize inhibitors prior to assay by RT-QuIC [43]. Briefly, 100 μl of saliva were diluted in 400 μl 1x PBS, 2 μl iron-oxide bead suspension were added, then incubated for 30 minutes on an end-over-end rotator. Samples were placed in a magnet, supernatants removed, the beads resuspended in 10 μl of 0.1% SDS, then assayed in quadruplicate at 2 μl /well. Three pools (n = 2 (+) pools, n = 1 (-) pool) of saliva from CWD-positive and CWD-negative deer were used as the saliva inocula for these studies. RT-QuIC seeding activity was used to determine the equivalent dose of the salivary inocula to concentrations of CWD-positive brain homogenate; expressed as ng of CWD-positive brain. Details are below.

**Fig 1:**
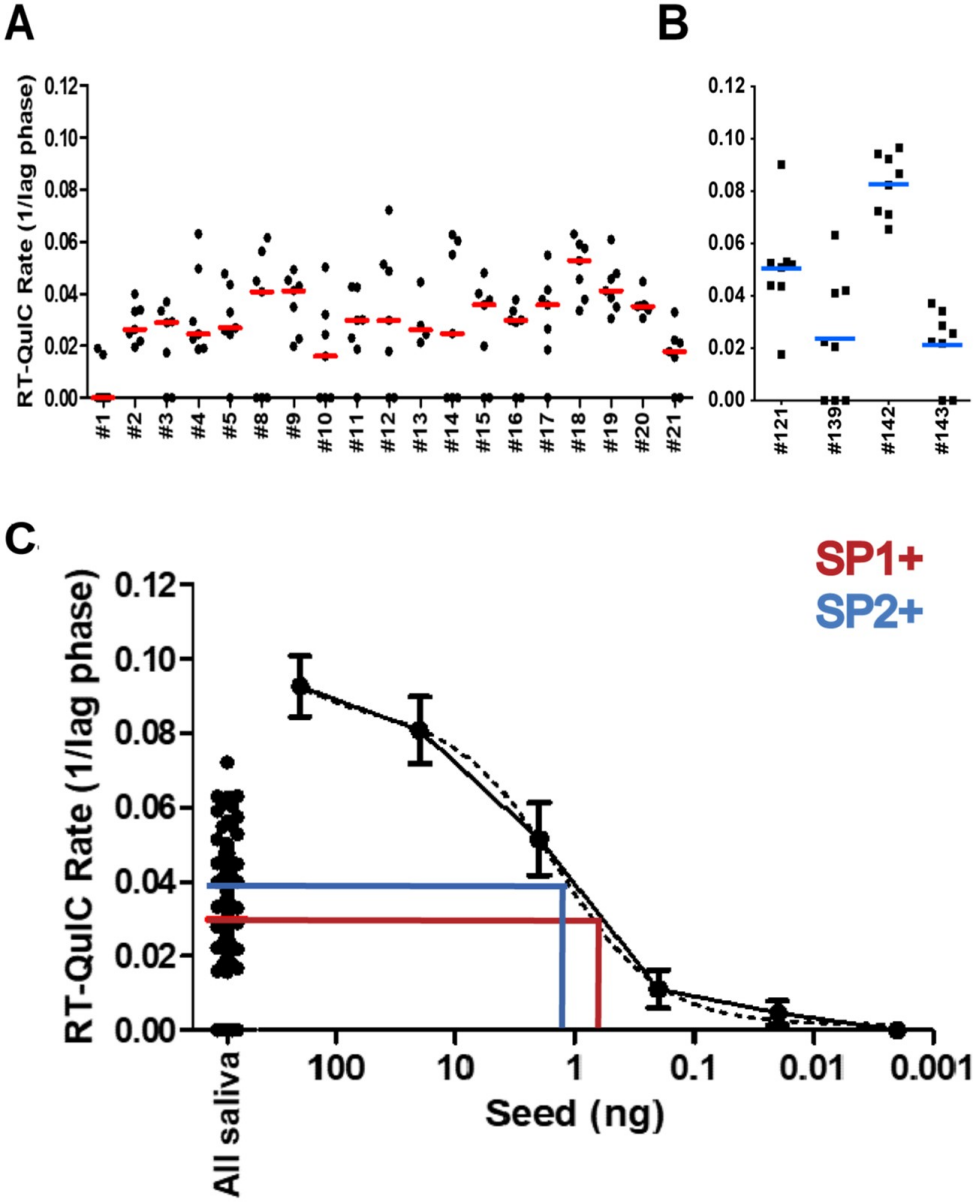
Estimation of prion seeding activity in pooled saliva inocula. (A) IOME-RT-QuIC prion seeding activity of 19 deer-saliva samples used to create a saliva inoculum pool 1 (SP1+). Individual saliva samples had varied prion seeding activity. (B) IOME-RT-QuIC prion seeding activity of saliva samples from 4 deer used to generate saliva inoculum pool 2 (SP2+). (C) Estimation of saliva inocula pools prion infectivity activity based on the mean rate of amyloid formation when compared with a standard curve of a CWD+ brain pool. Saliva pool 1 = 0.78 ng brain/100 μl (red line). Saliva pool 2 = 1.8 ng brain/100 μl (blue line).

Saliva Pool #1 (SP1+): We identified 19 positive saliva samples by IOME-RT-QuIC from CWD-infected white-tailed deer [11] throughout the course of disease with varied concentrations of prion seeding activity (Fig 1A). We used the amyloid formation rates from each saliva sample to calculate a weighted average for the pool based on the proportion each sample volume contributed to the total pool volume. The calculated saliva pool rate was compared to an RT-QuIC standard curve of brain homogenate CBP6 (Fig 1C; red), from which we extrapolated that 100 μl of the saliva pool was equivalent to ~0.78 ng CWD-positive brain wet weight. Thereby, we chose a 30 ml volume of the CWD-positive saliva pool for the inoculation dose that was equivalent to ~300 ng of CWD-positive brain.

Saliva Pool #2 (SP2+): We identified 4 positive saliva samples by IOME-RT-QuIC from infected white-tailed deer [11], collected at time of necropsy (Fig 1B). In this case, the samples were pooled, an amyloid formation rate was obtained, and the calculated saliva pool rate was compared to an RT-QuIC standard curve of brain pool CBP6. Again, we used the CBP6 brain homogenate titration curve to extrapolate that 100 μl of the saliva pool was equivalent to ~1.8 ng CWD-positive brain (Fig 1C; blue), and thereby a volume of 16.5 ml of the CWD-positive saliva pool was equivalent to ~300 ng of CBP6 brain.

Saliva Pool #3 (SP3-): Three [3] negative saliva samples from sham-inoculated white-tailed deer [11] were collected at time of necropsy, assayed by IOME-RT-QuIC, and determined to contain no seeding activity. All samples were pooled, assayed again, and confirmed negative by IOME-RT-QuIC. A volume of 16.5 ml was pooled and used as the matched negative control dose.

### Inoculation cohorts

Seven cohorts of deer (cohort #’s 1–7; n = 4 deer/cohort or n = 2 deer for the sham control cohort) were exposed to CWD inocula (brain or saliva) by *per os* (PO) instillation onto the oral mucous membrane lateral to the tongue. Inoculation was performed in a sufficiently light stage of anesthesia to ensure that the deer had an intact swallowing function. During the procedure each animal was manually supported and positioned with its head upright, facing forward, and parallel to the ground to ensure as natural a flow of oral fluids as attainable.

**Fig 2:**
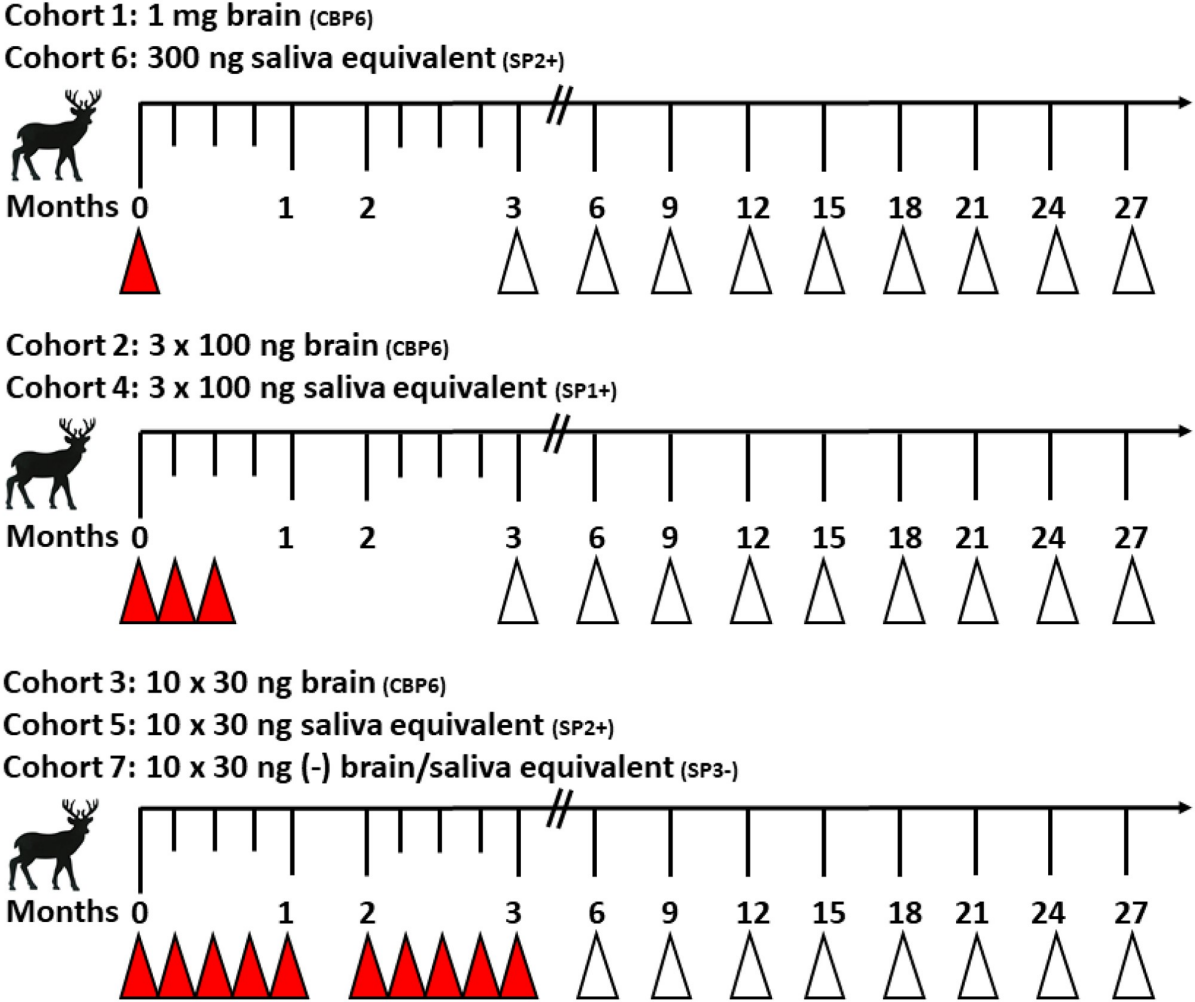
Experimental design of the current cohorts in this study. Solid triangles represent inoculation timepoints (0–12 weeks); open triangles represent longitudinal tissue biopsy collections to monitor for CWD (3–27 months).

The experimental design for the dosing of each cohort is in Fig 2. The dosage volumes and concentrations are in both Fig 2 and Table 1, and were as follows:

Cohort 1 (n = 4): 1 mg CWD-positive brain homogenate (CBP6), administered as a single dose in 1 ml 1XPBS (total dose = 1 mg brain pool CBP6);
Cohort 2 (n = 4): 300 ng CWD-positive brain homogenate (CBP6), administered as 3, 100 ng doses in 3 consecutive weeks (total dose = 300 ng);
Cohort 3 (n = 4): 300 ng CWD-positive brain homogenate (CBP6), administered as 10, 30 ng doses over 12 weeks. 30 ng doses were given once a week for 5 consecutive weeks, followed by a 4-week interval to ensure anesthesia safety, then resumed as weekly 30 ng doses for 5 consecutive weeks (total dose = 300 ng).
Cohort 4 (n = 4): 300 ng brain pool equivalent saliva (SP1+), administered as 3, 100 ng doses in 3 consecutive weeks. Each dose was contained in 10 ml of pooled saliva (total dose = 300 ng contained in 30 ml).
Cohort 5 (n = 4): 300 ng brain pool equivalent saliva (SP2+), administered as 10, 30 ng doses over 12 weeks. 30 ng doses were given once a week for 5 consecutive weeks, followed by a 4-week interval to ensure anesthesia safety, then resumed as weekly 30 ng doses for 5 consecutive weeks. Each dose was contained in 1.65 ml of pooled saliva (total dose = 300 ng contained in 16.5 ml).
Cohort 6 (n = 4): 300 ng brain pool equivalent saliva (SP2+), administered as a single 16.5 ml dose (total dose = 300 ng contained in 16.5 ml)
Cohort 7 (n = 2): 300 ng CWD-negative brain and 300 ng CWD-negative saliva, administered together as 10, 60 ng doses (30 ng each) over 12 weeks. 60 ng doses were given once a week for 5 consecutive weeks, followed by a 4-week interval to ensure anesthesia safety, then resumed as weekly 60 ng doses for 5 consecutive weeks. Each dose contained 30 ng CWD-negative brain contained in 3 ml - 1XPBS and 30 ng negative saliva contained in 1.65 ml (total dose = 600 ng contained in 46.5 ml).

**Table 1:** Study design summary of current and historical cohorts.

**Current Cohorts**	**n**	**Inocula**	**Total Dose (brain equiv.)**	**Inoculation Regimen**	**Attack rate %**	**MTTP (months)**	**Terminal**	
							**Clinical**	**Other**
1	4	Brain (CBP6)	1 mg	Single bolus (1 mg)	100	18.8	1	2
2	4	Brain (CBP6)	300 ng	3 x 100 ng; over 3 weeks	100	18.8	2	2
3	4	Brain (CBP6)	300 ng	10 x 30 ng; over 12 weeks	0	NA	0	4
4	4	Saliva (SP1+)	300 ng	3 x 100 ng; over 3 weeks	100	18	3	0
5	4	Saliva (SP2+)	300 ng	10 x 30 ng; over 12 weeks	0	NA	0	4
6	4	Saliva (SP2+)	300 ng	Single bolus (16.5 ml)	25	12	0	1
7	2	(-) Brain/Saliva (SP3-)	300 ng	10 x 30 ng; over 12 weeks	0	NA	0	2
**Historical Cohorts**	**n**	**Inocula**	**Dose**	**Inoculation Regimen**	**Attack rate %**	**MTTP (months)**	**Terminal**	
							**Clinical**	**Other**
8	6	(+) Brain	5–10 g	1-2g daily over 5 days	100	9.33	5	1
9	11	(+) Brain	1 g	Single bolus (1 g)	91	9.9	7	4
10	8	Brain (CBP6)	10 mg	Single bolus (1 ml)	87.5	10	8	0
11	6	(+) Saliva	50 ml	Administered over 7 days	83.3	14.4	1	5
12	2	(-) Brain/Saliva	10 g/50 ml	Administered over 14 days	0	NA	0	2

Each cohort was assigned a number for reference; n = the number of deer in each cohort; Inocula = the source of prion administered; Total dose = the total amount of inocula administered; Regimen = how inoculations were administered; Attack rate % = number of deer that became infected; MTTP = the mean time to positive (average in months of first tonsil IHC positive result for all deer); Terminal clinical = number of deer in each cohort euthanized due to clinical disease; Terminal other = number of deer euthanized due to other reasons; Terminal totals reflect only euthanized deer–deer not represented are still on study.

### Sample collections

Biopsies of tonsil and recto-anal lymphoid tissue (RAMALT) were collected every 3 months (Fig 2) and analyzed for prion seeding activity by RT-QuIC and CWD prion (PrP^CWD^) deposition by Immunohistochemistry (IHC) as described below. Deer were maintained for a minimum of 27 months post-inoculation (MPI) or until euthanasia due to progression of CWD.

### Historical CWD infection studies

Data from historical CWD infection studies in our laboratory were compared with the results of the present contemporary studies. These were performed under the same IACUC guidelines as above. All the deer had been inoculated orally with CWD brain or saliva as follows (Table 1):

Cohort 8 (n = 6): 5–10 g of CWD-positive whole brain [11, 30], administered as 1–2 g daily over 5 days.
Cohort 9 (n = 11): 1 g of CWD-positive brain administered as a single dose [20].
Cohort 10 (n = 8): 10 mg of CWD-positive brain homogenate (CBP6), administered as a single dose [25].
Cohort 11 (n = 6): 50 ml of saliva from CWD-positive deer (concentration unknown), administered daily over 7 days [11, 30].
Cohort 12 (n = 2): 10 g CWD-negative brain and 50 ml CWD-negative saliva administered over a 14-day period [11].

Tonsil and RAMALT biopsies were collected every 3 to 6 months post-inoculation. Cohorts 8, 9, 11, & 12 biopsies were analyzed for PrP^CWD^ detection by IHC only. Biopsies from Cohort 10 (10 mg) were analyzed by RT-QuIC for seeding activity and IHC for PrP^CWD^ deposition [44].

### Immunohistochemistry

**Fig 3:**
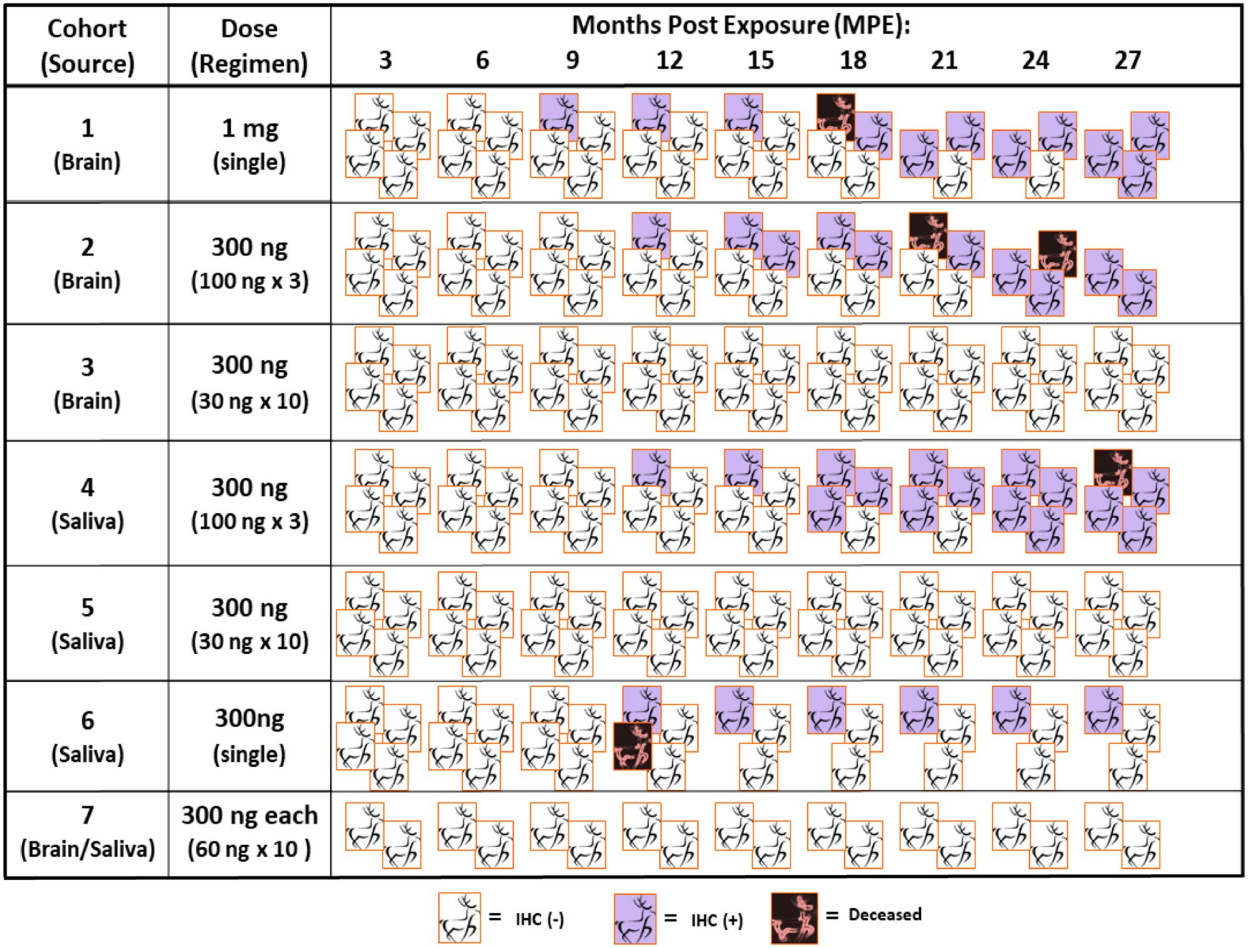
Longitudinal profile of tonsil biopsy results indicating when each deer in all 7 cohorts became IHC+ (purple box) or deceased (black box).

IHC remains the gold standard for detecting PrP^CWD^ and was therefore used to confirm infection and determine the mean time to positive (MTTP), defined as the average (in months) of the first IHC positive tonsil biopsy of all deer in each cohort, and could also be referred to as the mean incubation period (Fig 3, Table 1). Detection of PrP^CWD^ by IHC was performed using a modified version of a previously described protocol [7]. Briefly, formalin or PLP-fixed tissues were embedded in paraffin and 5μm sections mounted on positively charged glass slides. Sections were rehydrated through graded alcohols before treatment with 88% formic acid, followed by heat-induced epitope retrieval in citrate buffer. Tissues were treated with 3% hydrogen peroxide to quench endogenous peroxidase activity and blocked with TNB (0.1 M tris, 0.15 M sodium chloride, 0.5% blocking powder; Perkin Elmer) prior to overnight incubation with monoclonal antibody BAR-224 (1 mg/mL; Cayman Chemical) diluted 1:750. Envision+ System HRP (DAKO) was used for secondary detection with AEC substrate (ABCam) for visualization. No immunoreactivity was observed in negative control tonsil and RAMALT tissues that were tested simultaneously.

### Recombinant PrP substrate and preparation

Truncated recombinant Syrian hamster prion protein (rPrP), spanning codons 90–231 was produced, purified and refolded as originally described by the Caughey laboratory with modifications [31, 35]. Briefly, the cDNA was expressed in *E*. *coli* BL21-Star cells, inclusion bodies were harvested, then solubilized prior to binding to Ni-agarose resin (GE Healthcare Life Sciences). The recombinant prion protein was refolded, eluted, and dialyzed prior to aliquoting and storage at 4°C until use.

### RT-QuIC protocol

For each RT-QuIC reaction, we adapted the original protocol [35] to our current substrate mix [40, 42, 44], composed of 0.10 mg/ml rPrP, 10 μM thioflavin T (ThT), 320 mM NaCl, 1mM EDTA and 1X PBS, to which 96 μl were added to each well of a 96-well plate (Greiner Bio-One black, optical-bottom, VWR). Two [2] μl of each sample prepared with 0.1% sodium dodecyl sulfate (SDS) was then added to each well. A minimum of 8 replicates from each sample were assayed to determine average amyloid formation rate. RT-QuIC reactions were carried out with a fluorometer shaker (BMG Fluostar Omega^™^) programmed to alternate one minute shaking and rest cycles using double orbital shaking at 700 RPM. Thioflavin T fluorescence was recorded every 15 minutes at a 450 nm excitation wavelength, a 480 nm emission wavelength, and a gain of 1700. Experiments were conducted at 42°C for 62.5 hours. RT-QuIC data are displayed as 1/lag phase (a.k.a rate of amyloid formation/seeding activity). Lag phase was defined as the time in hours until a sample fluorescence reaches 5 standard deviations above the average baseline fluorescence.

## Results

### Very low doses of CWD prions are infectious to white-tailed deer

In attempt to better model the transmission of CWD in nature, we explored both the minimum oral infectious dose and potential cumulative dose effect of CWD prions from brain (central) or saliva (peripherally shed) origin. We thereby orally inoculated white-tailed deer with decreasing doses of either brain or saliva from CWD-positive deer as either: (a) a single dose (1 mg brain) or (b) 3 divided doses of 100 ng each (300 ng total) of brain or saliva administered one week apart. To study whether CWD infection may involve a cumulative/additive exposure phenomenon, we administered 300 ng of CWD-positive brain or saliva, divided as 10, 30 ng oral doses over 12 weeks. The results are described in accord with the successive series of inoculation cohorts listed in the methods section.

#### Brain-origin prions

We began by administering a 1 mg CWD-positive brain dose orally (10-fold lower dose than used in previous study [25]). In the next series we reduced that dose to 300 ng CWD-positive brain, divided into either 3 or 10 doses. The results follow.

**Fig 4:**
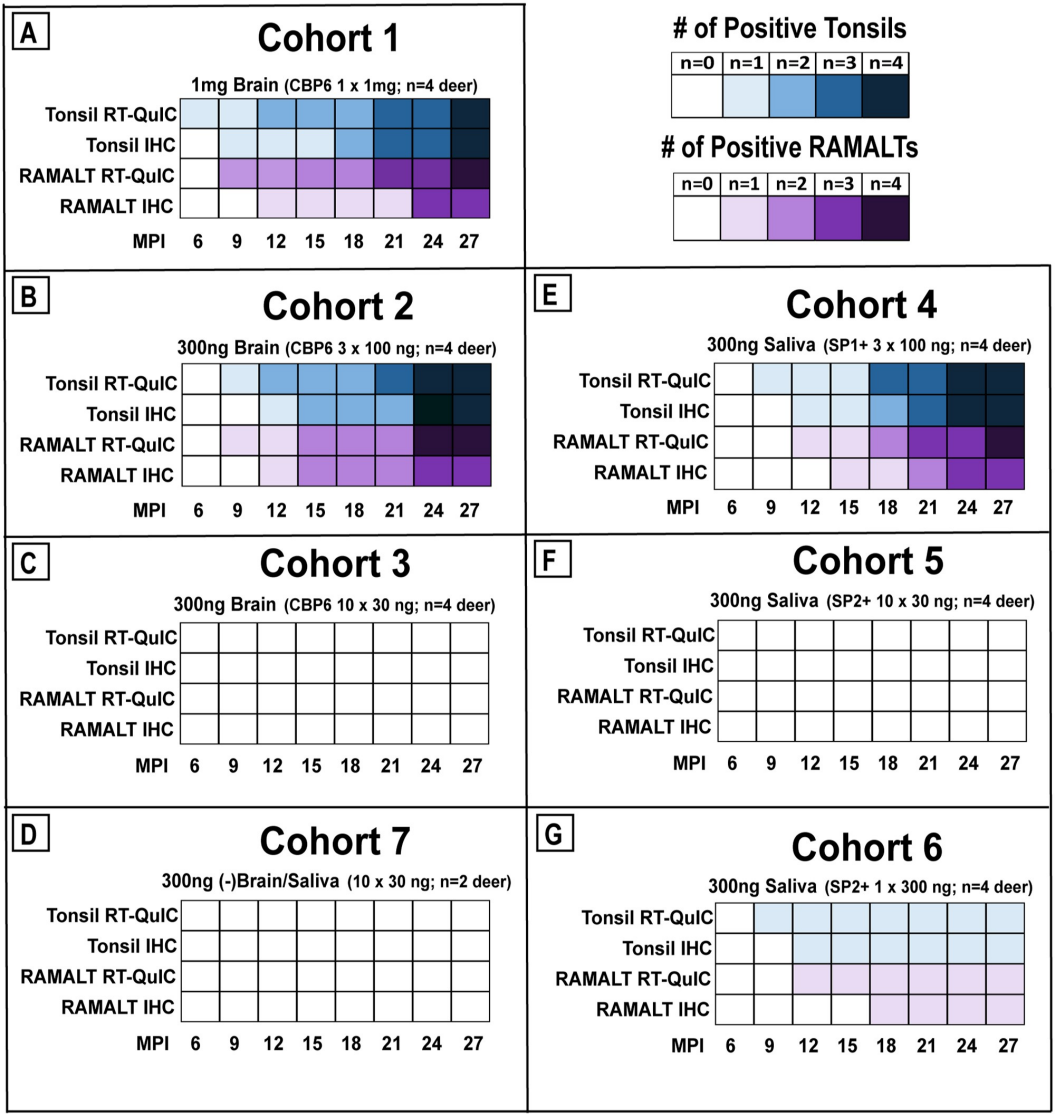
Cumulative gradients on tonsil (blue) and RAMALT (purple) biopsy positivity by RT-QuIC or IHC. Each block constitutes 8 time points when biopsies were collected and analyzed. Number of deer in each cohort is listed above the graph and MPI is on the x-axis.

Cohort 1 (1 mg brain, single dose): All deer developed CWD infection by 27 MPI as evidenced by clinical signs and/or PrP^CWD^ deposition in tissue biopsies (Fig 3). The mean time to positive (MTTP) in tonsil biopsies by IHC was 18.8 MPI [range: 9–27 MPI] with PrP^CWD^ deposition observed beginning at 9 MPI (Table 1, Figs 3 & 4A). RT-QuIC seeding activity was detected first in tonsil biopsies at 6 MPI (mean 14.3 MPI). In all four deer, RT-QuIC seeding activity was detected in RAMALT biopsies, as early as 9 MPI, with IHC positivity detected in 3 of the 4 animals (Fig 4A). One deer progressed to end-stage clinical disease and was euthanized at 18 MPI. Two deer were not clinically abnormal but were euthanized due to study housing limitations at 28 MPI (Table 1, Fig 3). One deer remains on study.

Cohort 2 (100 ng brain x 3 weekly doses): A similar pattern of results was observed to Cohort 1, i.e. a 100% infection rate by 21 MPI (Fig 3), an identical MTTP of PrP^CWD^ detection in tonsil at 18.8 MPI [range: 12–24 MPI], a similar pattern of RT-QuIC seeding activity (mean 15 MPI), and a similar timing pattern of PrP^CWD^ deposition in rectal biopsies beginning as early as 9 MPI (Table 1, Figs 3 & 4B). One deer in this cohort progressed to end-stage clinical disease and was euthanized at 21 MPI. Two deer were again not clinical but euthanized due to other circumstances at 24 & 28 MPI. The last deer became clinical and was euthanized at 38 MPI (Table 1).

Cohort 3 (30 ng brain x 10 weekly doses): The results differed markedly from cohorts 1 and 2. Over a longitudinal monitoring span of 27 MPI, none of the tonsil and RAMALT biopsies generated a positive result by RT-QuIC or IHC (Table 1, Figs 3 & 4C). All deer were necropsied and tissues examined (brain, retropharyngeal lymph nodes, tonsil, spleen, RAMALT) did not reveal a positive result in either assay.

Cohort 7 (10, 30 ng negative brain and 10, 30 ng saliva (SP3-)): All sham-inoculated control deer remained negative by IHC and RT-QuIC through the 27 month duration of study (Table 1, Figs 3 & 4D).

We correlated the codon 96 PRNP genotype to each deer’s first positive IHC result within the separate cohorts. In cohorts 1 & 2, three of four deer (75%) with PRNP 96GG displayed a positive result prior to 96GS deer [44]. Cohort 3 were all 96GG and all were negative. This agrees with previous studies that support genotypic differences [23, 41, 45].

The above results suggest that 100 to 300 ng of brain may be approaching the minimum oral infectious dose of CWD prions in white-tailed deer.

#### Saliva-origin prions

To test the hypothesis that prions shed in excreta may differ from those of brain origin, in regard to horizontal transmission, we sought to compare similar doses of saliva-origin prions to brain-origin prions above. We thereby administered 300 ng CWD-positive brain equivalent saliva doses divided and given as either 3 or 10 doses to respective cohorts. Additionally, we administered 300 ng CWD-positive equivalent saliva as a single bolus dose. The results were as follows:

Cohort 4 (100 ng brain equivalent saliva (SP1+) x 3 doses): The infectivity rate was 100% (Fig 3) by 24 MPI and the MTTP of tonsil biopsies by IHC was 18 MPI [range: 12–24 MPI]. PrP^CWD^ deposition was observed as early as 12 MPI, analogous to the cognate brain cohorts at the same or extrapolated equivalent dose (Table 1, Fig 3). RT-QuIC seeding activity was demonstrated in tonsil biopsies of all four animals, detected as early as 9 MPI (mean 17.3 MPI) (Figs 3 & 4E). All four RAMALT biopsies demonstrated RT-QuIC seeding activity, detected as early as 12 MPI, with three having positive IHC results detected as early as 15 MPI (Fig 4E). Here, three deer progressed to clinical disease and were euthanized at 27, 28, and 37 MPI (Table 1, Fig 3). One deer remains on study.
Cohort 5 (30 ng brain equivalent saliva (SP2+) x 10 doses): None of the assays to detect CWD infection (RT-QuIC or IHC on tonsil or RAMALT) were positive in any of the deer and no deer showed any clinical sign of CWD infection through 27 MPI (Table 1, Figs 3 & 4F). Necropsies were performed and all tissues (brain, retropharyngeal lymph nodes, tonsil, spleen, RAMALT) produced negative results in each of the assays.
Cohort 6 (300 ng brain equivalent saliva (SP2+) as single dose): In one of 4 deer (25% attack rate), RT-QuIC seeding activity was detected in tonsil and RAMALT tissues at 9 and 12 MPI respectively. In the same deer, IHC positivity was detected in tonsil tissue at 12 MPI (Fig 3) and in the RAMALT at 18 MPI (Fig 4G). One deer in this cohort died unexpectedly of undetermined causes just after 9 MPI; multiple terminal tissues collected from this animal were negative by RT-QuIC and IHC analysis. The remaining two deer were negative throughout the 27-month study period (Table 1, Figs 3 & 4G) and remain on study.
Cohort 7 (10, 30 ng CWD-negative brain and 10, 30 ng saliva (SP3-)): All sham-inoculated control deer remained negative for evidence of CWD infection by all assays throughout the course of the 27-month study (Table 1, Figs 3 & 4D).

The codon 96 PRNP genotypes of the deer in above groups were assessed (cohorts 4 and 6). Four of six deer (67%) of PRNP 96GG became CWD+ vs. one of the two 96GS deer, and all four 96GG deer developed first indication of CWD infection before 96GS deer in their respective cohorts [44]. Cohort 5 (96GG (n = 3); 96GS (n = 1)) were all negative. These results are consistent with previous studies indicating slower disease kinetics in 96GS vs. 96GG deer [23, 41, 45].

The above results from oral exposure of deer to saliva estimated to be equivalent to 300 ng of brain-origin prion seeding activity again suggest that this dose exposure approaches the minimum CWD oral infectious dose whether the prion inoculum was of brain or saliva origin.

### Historical studies have substantially exceeded minimum infectious dose

We compared previous deer studies in our laboratory that employed oral CWD exposure to examine the relationship between dose, infection, and genotype.

#### Brain-origin prions

**Fig 5:**
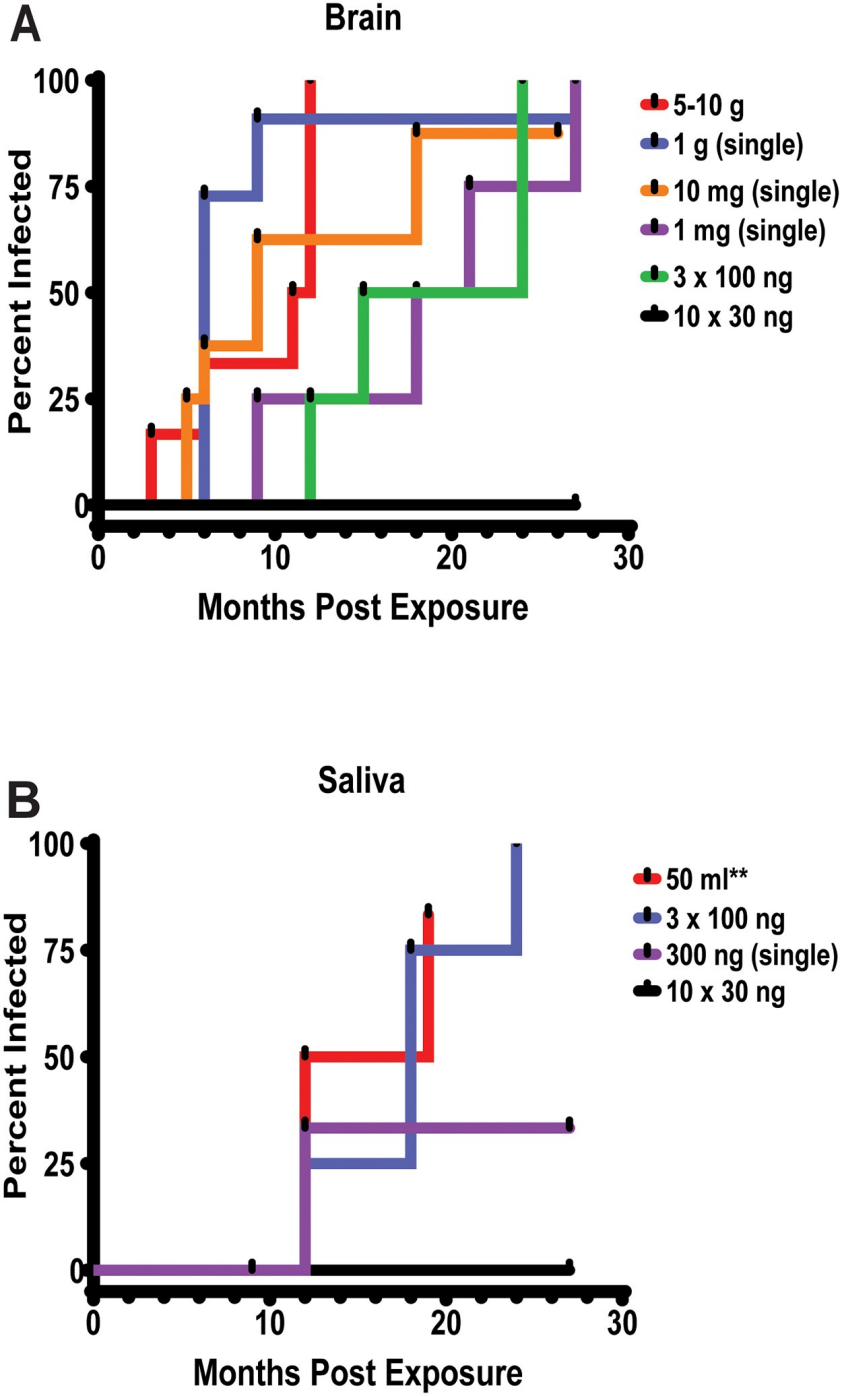
Infection rates expressed by first IHC(+) tonsil biopsy in white-tailed deer orally exposed to varying doses of CWD prions of (A) brain or (B) saliva origin; 50 mL** = unknown concentration administered.

Cohorts 8–10: Oral administration of 10 mg to 10 g of CWD-positive brain over 1 to 5-day periods [11, 20, 25, 30] produced MTTP’s ranging from 9.3 to 10 MPI with attack rates of 87.5 to 100% by 27 MPI (Table 1, Fig 5A).

Cohorts 1, 2 above: 1 mg and 300 ng CWD-positive brain administered as either a single dose or 3 divided doses produced a 100% infectivity rate but MTTP increased to 18.8 MPI (Table 1, Fig 5A).

Cohort 3 above: When the same 300 ng CWD-positive brain was administered as 10, 30 ng doses over 12 weeks, the attack rate dropped to 0% (Table 1, Fig 5A).

Within the historical cohorts, 21 of 25 deer (84%) were IHC positive by 12 MPI. When we compared the association of first IHC positive result to genotype, cohorts 8 had all three 96GG deer positive before the three 96GS deer, whereas cohort 9 had four 96GG deer positive at the same time as all four 96GS deer [20]. Cohort 10 consisted of all 96GG deer, thus precluding genotype comparisons. The results aligned with the current studies (see above), with the exception that the MTTPs were delayed in the low dose cohorts.

Overall the results of the above studies demonstrate an inverse correlation between dose and mean time to first positive and suggest a minimum infectious dose ranging from 100–300 ng CWD-positive brain.

#### Saliva-origin prions

Cohort 11: 50 ml of pooled CWD-positive saliva was administered over 7 days [11, 30] produced a 91% attack rate and a MTTP of 14.8 MPI (Fig 5B; Table 1). Prion seeding activity/concentration of this inoculum is unknown as no samples remain for testing.

Cohort 4 above: 30 ml of saliva pool SP1+ (equivalent to ~300 ng of CWD-positive brain) administered over 3 weekly doses produced a 100% attack rate with an MTTP of 18 MPI (Fig 5B; Table 1).

Cohort 5 above: 16.5 ml of saliva pool SP2+ (equivalent to ~300 ng of CWD-positive brain) administered as 10 doses over 12 weeks produced a 0% attack rate (Fig 5B; Table 1).

Cohort 6 above: 16.5 ml of saliva pool SP2+ (equivalent to ~300 ng of CWD-positive brain) administered as a single bolus dose produced a 25% attack rate (Fig 5B; Table 1).

Comparisons of historical saliva inoculation studies to the current study demonstrated a similar pattern to brain inocula, suggesting that dose is directly related to MTTP and that 300 ng brain equivalent material approaches the minimum infectious dose. The results also suggest infectivity of brain vs. saliva prions is similar. As all historical Cohort 11 deer were 96GG, no genotype comparisons could be made. Moreover, these results indicate that (our) historical CWD infection studies using CWD-positive brain substantially exceeded the challenge needed to initiate infection by up to a million-fold.

## Discussion

As CWD expands across North America and Scandinavia, how this disease is transmitted so efficiently remains unclear, given the low concentrations of prions shed in secretions and excretions [13, 14]. The present studies demonstrated that a single oral exposure to as little as 300nmg of CWD-positive brain or equivalent saliva can initiate infection in 100% of exposed white-tailed deer. However, distributing this dose as 10, 30 ng exposures failed to induce infection. Overall, these results suggest that the minimum oral infectious exposure approaches 100 to 300 ng of CWD-positive brain equivalent. These dynamics also invite speculation as to whether potential infection co-factors, such as particle binding [46, 47] or compromises in mucosal integrity may influence infection susceptibility, as suggested from two studies in rodent models [48, 49].

Few studies in rodent models have explored oral infection with murine or hamster adapted scrapie by assessing the same total dose administered as a single bolus vs. the same bolus divided into fractional, sequential exposures [50–52]. The results reported by Diringer et al. [50] and Jacquemot et al. [52] have indicated that divided-dose exposures were as effective as a single bolus only if the interval between doses was short (1–2 days). In deer, we likewise found that when a total dose of 300 ng of brain was administered as 10 doses divided doses over 12 weeks this exposure failed to induce CWD infection, whereas three weekly 100 ng doses (300 ng total) induced infection. While this latter outcome may have involved an additive dynamic, we cannot exclude that a dose 100 ng alone also may have been sufficient to establish infection. Our conclusions here are unfortunately limited by the absence of a single 100 ng dose group. Additional experiments are needed to further directly compare single vs. divided exposures to strengthen the tenet that establishment of CWD infection is more a threshold than cumulative dose phenomenon.

We also sought to examine a relatively unexamined possibility that prions emanating from different tissues and/or cells may possess different capacities to establish infections by mucosal routes. Our results indicated that brain and saliva inocula containing similar levels of prion seeding activity, also had similar infectivity, which did not support our hypothesis that saliva prions may be more infectious by mucosal routes. There are of course, several caveats bearing on this conclusion. These could include: the inherent limits in using an *in vitro* seeding assay as a surrogate to equate *in vivo* infectivity, the likelihood that small differences in prion susceptibility among deer may be more significant at very low exposure doses, and the greater variation of inoculum uptake and routing through mucosal surfaces associated with the oral route of exposure.

The chief correlate we observed between magnitude of infectious dose and disease course was in time from exposure to first detected amplification of prions in tonsil, an event which is closely followed by or concurrent with detection in pharyngeal lymph nodes [41]. Once a threshold dose was established, the subsequent pathogenesis of infection and disease appeared to vary little.

In addition to potential cofactors that could influence CWD infectivity, such as particle binding [47] and compromised mucosal integrity [48, 53], there is PRNP genotype, in which polymorphisms at codon 96 of the white-tailed deer are known to affect the temporal dynamics of CWD infections [23, 41, 45]. In the present studies, most cohorts of 96GG deer became CWD-positive before 96GS animals in the same exposure group [cohorts 1, 2, 4, 6]. Thus, the low dose studies are consistent with the current concept of delayed conversion rate in PRNP 96GS vs. 96GG white-tailed deer [44].

In conclusion, we have attempted to model and better understand CWD infection relative to natural exposure. The results demonstrate: (a) that the minimum CWD oral infectious dose is vastly lower than historical studies used to establish infection; (b) that a direct relationship exists between dose and incubation time to first prion replication detection in tonsils, irrespective of genotype; (c) that a difference was not discernible between brain vs. saliva source prions in ability to establish infection or in resultant disease course; and (d) that the CWD infection process appears to conform more to a threshold dose than an accumulative dose dynamic.
