# Correction: Very low oral exposure to prions of brain or saliva origin can transmit chronic wasting disease

**DOI:** 10.1371/journal.pone.0253356

**Published:** 2021-06-10

**Authors:** The PLOS ONE Staff

The following information is missing from the Funding statement: EAH: RO1-NS-061902, PO1-AI-077774, T32-OD-010437 (National Institutes of Health), CKM: R01-NS-076894 (National Institutes of Health). The publisher apologizes for the error.

## References

[1] Denkers ND, Hoover CE, Davenport KA, Henderson DM, McNulty EE, Nalls AV, et al. (2020) Very low oral exposure to prions of brain or saliva origin can transmit chronic wasting disease. PLoS ONE 15(8): e0237410. 10.1371/journal.pone.0237410 32817706 PMC7446902

